# Shiga Toxin-Producing *Escherichia coli* in Yaks (*Bos grunniens*) from the Qinghai-Tibetan Plateau, China

**DOI:** 10.1371/journal.pone.0065537

**Published:** 2013-06-11

**Authors:** Xiangning Bai, Ailan Zhao, Ruiting Lan, Youquan Xin, Hui Xie, Qiong Meng, Dong Jin, Bo Yu, Hui Sun, Shan Lu, Jianguo Xu, Yanwen Xiong

**Affiliations:** 1 Collaborative Innovation Center for Diagnosis and Treatment of Infectious Diseases, State Key Laboratory for Infectious Disease Prevention and Control, National Institute for Communicable Disease Control and Prevention, Changping, Beijing, China; 2 School of Biotechnology and Biomolecular Sciences, University of New South Wales, Sydney, NSW, Australia; 3 Qinghai Institute for Endemic Disease Prevention and Control, Xining, Qinghai Province, China; Northeast Agricultural University, China

## Abstract

Shiga toxin (Stx)-producing *Escherichia coli* (STEC) are recognized as important human pathogens of public health concern. Many animals are the sources of STEC. In this study we determined the occurrence and characteristics of the STEC in yaks (*Bos grunniens*) from the Qinghai-Tibetan plateau, China. A total of 728 yak fecal samples was collected from June to August, 2012 and was screened for the presence of the *stx*_1_ and *stx*_2_ genes by TaqMan real-time PCR after the sample was enriched in modified Tryptone Soya Broth. Of the 138 (18.96%) *stx*_1_ and/or *stx*_2_-positive samples, 85 (61.59%) were confirmed to have at least 1 STEC isolate present by culture isolation, from which 128 STEC isolates were recovered. All STEC isolates were serotyped, genotyped by pulsed-field gel electrophoresis (PFGE) and characterized for the presence of 16 known virulence factors. Fifteen different O serogroups and 36 different O:H serotypes were identified in the 128 STEC isolates with 21 and 4 untypable for the O and H antigens respectively. One *stx*_1_ subtype (*stx*_1a_) and 5 *stx*_2_ subtypes (*stx*_2a_, *stx*_2b_, *stx*_2c_, *stx*_2d_ and *stx*_2g_) were present in these STEC isolates. Apart from *lpfA*_O157/OI-141_, *lpfA*_O157/OI-154_, *lpfA*_O113_, *katP* and *toxB* which were all absent, other virulence factors screened (*eaeA*, *iha*, *efa1*, *saa*, *paa*, *cnf1*, *cnf2*, *astA, subA*, *exhA* and *espP*) were variably present in the 128 STEC isolates. PFGE were successful for all except 5 isolates and separated them into 67 different PFGE patterns. For the 18 serotypes with 2 or more isolates, isolates of the same serotypes had the same or closely related PFGE patterns, demonstrating clonality of these serotypes. This study was the first report on occurrence and characteristics of STEC isolated from yaks (*Bos grunniens*) from the Qinghai-Tibetan plateau, China, and extended the genetic diversity and reservoir host range of STEC.

## Introduction

Shiga toxin-producing *Escherichia coli* (STEC) are recognized globally as major food-borne pathogens. Clinical manifestations of STEC infections in humans range from non-bloody diarrhea to hemorrhagic colitis (HC) and sometimes fatal hemolytic uremic syndrome (HUS) complications. There are more than 200 known STEC serotypes associated with human illness [1]. O157:H7 is the most frequently encountered STEC in human infections [2]. Many outbreaks and sporadic infections caused by STEC O157:H7/NM have been reported in different regions of the world [3], [4], [5], [6], [7], [8]. However, non-O157 STEC isolates have been increasingly associated with human infections and outbreaks. In 2011, Germany experienced the largest outbreak of non-O157 STEC, O104:H4, ever recorded with 3,816 cases including 845 HUS cases and 54 deaths, similar outbreaks were reported in France and other counties in Europe subsequently [9], [10], [11], [12]. Non-O157 STEC infections are likely to be under-reported due to awareness and difficulties in isolation and identification in clinical laboratories.

STEC possesse a number of virulence factors, with the production of Shiga toxins (Stxs) being the most critical which leads to the damage of the endothelial cells and potential HUS [13]. The Stx family can be categorized into two major types, Stx1 and Stx2 [14], which differ in their effects on the endothelial cells [15]. Stx1 and Stx2 are further divided into 3 subtypes (Stx1a, Stx1c and Stx1d) and 7 subtypes (Stx2a to Stx2g) respectively [14]. The different Stx types and/or subtypes may be associated with differences in the severity of illness [16], [17]. Other factors are purported to increase virulence in STEC isolates. Cytotoxic necrotizing factor 1 (CNF1) and its isoform CNF2 are cytotoxins that activate Rho GTPases leading to tissue damage, perturb the epithelial barrier and impair the function of immune cells [18]. EAST-1 is a genetically distinct toxin structurally related to heat-stable enterotoxin (STa) of enterotoxigenic *E. coli* [19]. Subtilase cytotoxin (SubAB) is the prototype of a new AB_5_ toxin family produced by a subset of STEC strains [20]. SubAB is lethal for mice and induces pathological features overlapping those seen in HUS [21].

Typically STEC also possesse the locus of enterocyte effacement (LEE), which encodes proteins necessary for the formation of attaching and effacing (A/E) lesions including the intimin, a translocated intimin receptor (Tir), a type III secretion apparatus, and effector proteins translocated by the secretion system [22]. In the absence of intimin, other adherence factors may increase adherence and virulence in STEC. These include Iha (IrgA homologue adhesin) which is a STEC adherence-conferring molecule conferring the adherence phenotype upon nonadherent laboratory *E. coli* [23]; Efa1 (EHEC factor for adherence 1) which was shown to be essential for the adherence of the bacteria to cultured epithelial cells, hemagglutination and autoaggregation [24]; LPF (long polar fimbriae) which is closely related to the LPF of *Salmonella enterica* serovar Typhimurium [25]; and Saa (STEC autoagglutinating adhesin) which is an autoagglutinating adhesin produced by LEE-negative STEC strains [26]. Paa (porcine A/E associated protein), which was first discovered in porcine enteropathogenic *E. coli*, contributes to the early stages of the development of the A/E lesions and is also present in O157:H7 [27]. Additionally, many STEC strains contain the heterologous 60-MDa virulence plasmid, which contains a number of virulence genes: an enterohemolysin (*ehxA*), a catalase-peroxidase (*katP*), an extracellular serine protease (*espP*) and a adhesin (*toxB*) [28].

Domestic or wild animals are the primary sources of STEC, such as cattle, pig, sheep, dog, cat, horse, deer and wild boars [29], [30], [31], [32], [33], with cattle being regarded as the main natural reservoirs [34]. Humans are the accidental host of STEC through the ingestion of contaminated meat, milk, vegetables, fruits and water. The yak (*Bos grunniens*) lives at high altitude (above 3,000 m) in China, India, Nepal and other countries. There are more than 14 million yaks on the Qinghai-Tibetan plateau, which represent more than 90% of the world yak population. Yaks are adapted to the harsh environments of severe cold, less atmospheric oxygen, strong ultra-violet radiation and poor forage resources. Domestic yaks are of economic importance (such as meat, milk for food, hide for leather and dung for fuel) to Tibetans and other nomadic pastoralists in high-altitude environments [35], [36]. In this study we determined the occurrence and characteristics of STEC from yaks from the Qinghai-Tibetan plateau, China.

## Materials and Methods

### Collection of Samples and Enrichment of Fecal Samples

The investigation was carried out in Yushu tibetan autonomous prefecture, Qinghai province, China. Four big herds (more than one thousand free-ranging yaks) were chosen during June to August, 2012. The sites are Jielachong (3,970 m above msl (mean sea level), latitude of 33°48′ and longitude of 96°51′), Gandacun (4,322 m above msl, latitude of 33°13′ and longitude of 96°73′), Batangtan (3,987 m above msl, latitude of 32°51′ and longitude of 96°56′) and Batang (3,871 m above msl, latitude of 32°84′ and longitude of 97°11′) respectively. Fresh fecal samples of yaks were collected in 2 ml sterile tubes containing Luria-Bertani (LB) medium (30% glycerol added). Collected samples were stored at −20°C immediately and transported to the laboratory in National Institute of Communicable Disease Control and Prevention, China CDC in ice cold conditions. A total of 728 fecal samples was collected for the present study.

Each fecal sample was inoculated into modified Tryptone Soya Broth (mTSB) supplemented with novobiocin (10 µg/µl) (Oxoid, UK) and incubated at 37°C for 18 to 24 h with shaking at 200 rpm.

### *stx* Screening by TaqMan Real-time PCR

The enriched samples were investigated for *stx*_1_ */stx*_2_ genes by TaqMan duplex real-time PCR assay developed in this study (Probe-1, primers Stx1Fr and Stx1Rr for *stx*_1_; Probe-2, primers Stx2Fr and Stx2Rr for *stx*_2_) (**Table 1**). Briefly, 1.5 ml of each enrichment sample was centrifuged at 13,000×g for 2 min, the pellet was suspended in 150 µl of the rapid lysis buffer (100 mM NaCl, 10 mM Tris-HCl [pH 8.3], 1 mM EDTA [pH 9.0], 1% Triton X-100), then boiled for 10 min, and centrifuged at 13,000×g for 2 min. The supernatant was then used as template. Real-time PCR was performed with the Rotor-Gene Q Real-Time PCR system (Qiagen, Germany) using oligonucleotide primers and fluorescent probes targeting *stx*_1_ and *stx*_2_. The amplification conditions were as follows: initial denaturation at 95°C for 10 s and then 40 cycles of 95°C for 5 s and 60°C for 20 s.

**Table 1:** PCR primers used for the detection of STEC virulence or adherence genes.

Target	Primer	Oligonucleotide sequence (5′-3′)	Amplicon size (bp)	Annealing temperature (°C)	Reference
*stx*_1_	Stx1Fr	TGGATTTAATGTCGCATAGTGGAA	82	60	This study^a^
	Stx1Rr	CAGCTGTCACAGTAACAAACCGTAA			
	Probe-1	HEX-CACTGACGCAGTCTGTGGCAAGAGC-BHQ1			
*stx*_2_	Stx2Fr	CAACGGACAGCAGTTATACCACTCT	103	60	This study^a^
	Stx2Rr	TTAACGCCAGATATGATGAAACCA			
	Probe-2	FAM-CCGGAATGCAAATCAGTCGTCACTCA-BHQ1			
*stx*_1_	Stx1F	AAATCGCCATTCGTTGACTACTTCT	370	58	This study^b^
	Stx1R	TGCCATTCTGGCAACTCGCGATGCA			
*stx*_2_	Stx2F	CAGTCGTCACTCACTGGTTTCATCA	283	58	This study^b^
	Stx2R	GGATATTCTCCCCACTCTGACACC			
*stx*_1_	SltIF	TCGCATGAGATCTGACC	1470	60	[56] c
	SltIR	AACTGACTGAATTGAGATG			
*stx*_2_	GK1	ATGAAGTGTATATTATTTAAATGG	1260	55	[56] c
	GK4	TCAGTCATTATTAAACTGCAC			
*eaeA*	eaeAF	TCAATGCAGTTCCGTTATCAGTT	482	58	[57]
	eaeAR	GTAAAGTCCGTTACCCCAACCTG			
*iha*	iha-I	CAGTTCAGTTTCGCATTCACC	1305	56	[58]
	iha-II	GTATGGCTCTGATGCGATG			
*efa1*	efa1F	GAGACTGCCAGAGAAAG	479	51	[24]
	efa1R	GGTATTGTTGCATGTTCAG			
*lpfA*_O157/OI-154_	OI-154F	GCAGGTCACCTACAGGCGGC	525	55	[59]
	OI-154R	CTGCGAGTCGGCGTTAGCTG			
*lpfA*_O157/OI-141_	OI-141F	CTGCGCATTGCCGTAAC	412	54	[60]
	OI-141R	ATTTACAGGCGAGATCGTG			
*lpfA*_O113_	O113F	ATGAAGCGTAATATTATAG	573	52	[61]
	O113R	TTATTTCTTATATTCGAC			
*saa*	saaF	CGTGATGAACAGGCTATTGC	119	52	[62]
	saaR	ATGGACATGCCTGTGGCAAC			
*paa*	M155-F1	ATGAGGAAACATAATGGCAGG	350	60	[63]
	M155-R1	TCTGGTCAGGTCGTCAATAC			
*cnf1*	CNF1-fp	GGCGACAAATGCAGTATTGCTTGG	552	63	[64]
	CNF1-bp	GACGTTGGTTGCGGTAATTTTGGG			
*cnf2*	CNF2-fp	GTGAGGCTCAACGAGATTATGCACTG	839	63	[64]
	CNF2-bp	CCACGCTTCTTCTTCAGTTGTTCCTC			
*astA*	EAST11a	CCATCAACACAGTATATCCGA	111	55	[65]
	EAST11b	GGTCGCGAGTGACGGCTTTGT			
*subA*	SubHCDF	TATGGCTTCCCTCATTGCC	556	65/60	[66]
	SubSCDR	TATAGCTGTTGCTTCTGACG			
*ehxA*	hlyAF	GGTGCAGCAGAAAAAGTTGTAG	1551	57	[67]
	hlyAR	TCTCGCCTGATAGTGTTTGGTA			
*katP*	wkat-B	CTTCCTGTTCTGATTCTTCTGG	2125	56	[68]
	wkat-F	AACTTATTTCTCGCATCATCC			
*espP*	esp-A	AAACAGCAGGCACTTGAACG	1830	56	[28]
	esp-B	GGAGTCGTCAGTCAGTAGAT			
*toxB*	toxB.911F	ATACCTACCTGCTCTGGATTGA	602	55	[69]
	toxB.1468R	TTCTTACCTGATCTGATGCAGC			

a Primers and probes used for TaqMan real-time PCR for the screening of *stx*_1_ and *stx*_2_.

b Primers used for duplex PCR for the detection of *stx*_1_ and *stx*_2_.

c Primers used for amplifying and sequencing the full length of *stx*_1_ or *stx*_2_.

The performance of the TaqMan real-time PCR was validated with reference plasmid constructs, pMD18-stx1 and pMD18-stx2, containing a copy of *stx*_1_ and *stx*_2_ respectively and O157:H7 EDL933 spiked human stools. The limit of detection for *stx*_1_ or *stx*_2_ was 1×10^2^ copies per reaction using the reference plasmids and 3.55×10^3^ CFU per gram in spiked human stools. Seventeen non-STEC pathogens were used to evaluate assay specificity, including enteroaggregative *E. coli*, enteroinvasive *E. coli*, enteropathogenic *E. coli*, enterotoxigenic *E. coli*, *Shigella flexneri*, *Shigella sonnei*, *Listeria monocytogenes*, *Yersinia enterocolitica*, *Yersinia pseudotuberculosis*, *Vibrio cholerae*, *Salmonella typhi*, *Salmonella typhimurium*, *Vibrio parahaemolyticus*, *Staphylococcus aureus*, *Aeromonas hydrophila*, *Citrobacter freundii* and *Campylobacter jejuni*. No false-positives were observed.

### Isolation of Shiga Toxin-producing *Escherichia coli*

Enriched fecal samples tested positive by the TaqMan real-time PCR assay for *stx*_1_ and/or *stx*_2_ genes were plated onto CHROMagar™ ECC agar (CHROMagar, Paris, France ), and incubated at 37°C overnight. Ten presumptive colonies (blue or colorless, round moist colonies, but the colonial morphology may be variable) on each plate were picked and screened for the presence of *stx*_1_ and/or *stx*_2_ genes by single colony duplex PCR assay (primers Stx1F and Stx1R for *stx*_1_, primers Stx2F and Stx2R for *stx*_2_) (**Table 1**). The *stx*-positive colonies were plated onto LB and incubated overnight to obtain single colonies for further identification. If all 10 colonies were negative for *stx*, another 10 colonies were picked and screened. Finally, 1 to 3 *stx*-positive isolates from each sample were collected for further investigation.

### Biochemical Test and Serotyping of STEC Isolates

*stx*-positive isolates were confirmed to be *E. coli* by biochemical identification using the API 20E system (bioMérieux, France). The O serogroups were screened by PCR using O antigen specific primers in DebRoy *et al*. [37]. *E. coli* O antisera (Statens Serum Institute, Denmark) were used to confirm the O group PCR results. The H type of each isolate was determined by amplifying and sequencing the *fliC* gene and comparing sequences in GenBank as previously described [34].

### Identification of Virulence and Adherence Factor Genes

All STEC isolates were subjected to PCR for detection of intimin-encoding gene (*eaeA*), putative adhesin genes (*iha*, *efa1*, *lpfA*_O157/OI-141_, *lpfA*_O157/OI-154_, *lpfA*_O113_, *saa*, *paa*), virulence-associated genes (*cnf1*, *cnf2*, *astA*, *subA*), the large heterologous virulence plasmid genes (*exhA*, *katP*, *espP*, *toxB*) using primers listed in **Table 1**.

### *stx* Subtyping

Genotyping of s*tx*_1_ and *stx*_2_ subtypes was conducted by the PCR subtyping method developed by Scheutz *et al*. [14]. The complete *stx*_1_ and/or *stx*_2_ genes of some STEC isolates were amplified (primers SltIF and SltIR for *stx*_1_, primers GK1 and GK4 for *stx*_2_) (**Table 1**) and sequenced. DNA sequences were then analyzed and compared with the published sequences of *stx*_1_ and *stx*_2_ subtypes in the GenBank.

### Pulsed-field Gel Electrophoresis (PFGE)

Pulsed-field gel electrophoresis was performed using the non-O157 STEC subtyping protocol from PulseNet, USA with some modifications. The genomic DNA was digested with 45 U of *Xba*I (Takara, Dalian, China) at 37°C for 2 h. A contour-clamped homogenous electric field apparatus CHEF-Mapper (Bio-Rad, USA) was used. The pulse time was ramped from 6.76 s to 35.38 s over 18 h at 6.0 V/cm. The image was captured with a Gel Documentation 2000 software (Bio-Rad, USA) and exported to Bionumerics (Version 4.0, Applied Maths BVBA, Belgium) for analysis of the PFGE patterns. An UPGMA dendrogram was drawn using the BioNumerics software.

### Ethics Statement

Fecal samples of free-ranging yaks were acquired with the consent of the owners of the lands and animals. The study was approved by the ethics committee of National Institute for Communicable Disease Control and Prevention, China CDC, according to the medical research regulations of Ministry of Health, China.

## Results

### Prevalence of STEC in Yak Fecal Samples

Out of 728 yak fecal samples analyzed in this study, 138 (18.96%) were positive for *stx*_1_and/or *stx*_2_ genes using TaqMan real-time PCR assay. The four herds showed different *stx*_1_ and/or *stx*_2_ positive rates ranging from 14% to 29%. One hundred and twenty eight STEC isolates were isolated from 85 of the 138 *stx* positive fecal samples giving a culture positive STEC rate of 61.59% for *stx* PCR positive samples and 11.68% for all samples (**Table 2**). A single isolate was obtained from 44 fecal samples, two isolates per sample were recovered from 39 fecal samples, and three isolates each were obtained from two samples.

**Table 2:** Prevalence of Shiga toxin-producing *Escherichia coli* in yaks.

Herd	No. of samples	No. of *stx* positive (%)	No. of samples with STEC isolates (%)	No. of STEC isolates (%)
1	200	28 (14)	23 (11.5)	40 (20)
2	200	58 (29)	31 (15.5)	40 (20)
3	100	15 (15)	3 (3)	5 (5)
4	228	37 (16.23)	28 (12.28)	43 (18.86)
Total	728	138 (18.96)	85 (11.68)	128 (17.58)

### Serogroups and Serotypes

In total, 15 different O serogroups and 12 different H types were identified in the 128 STEC isolates, which belonged to 36 divergent serotypes namely O2:H21, O2:H45, O6:H14, O6:H21, O6:Hnt, O8:H2, O8:H9, O8:H16, O8:H19, O8:H45, O12:H12, O22:H8, O52:H2, O66:H8, O66:H21, O78:H8, O78:H21, O78:H44, O78:H45, O78:Hnt, O12/O78:H44, O117:H2, O117:H21, O123:H8, O127:H8, O137:H21, O149:H45, O158:H8, O158:H16, O165:H8, O165:H21, Ont:H7, Ont:H8, Ont:H21, Ont:H40, Ont:H44. Twenty one isolates were untypable for O groups and 4 isolates were untypable for H antigen as there was no product from the PCR amplification of the *fliC* gene. The predominant serotypes were O8:H16, O2:H45, O117:H21, O78:H8, O8:H9, Ont:H8, Ont:H21, and O78:H45 which consisted of 14 (10.94%), 14 (10.94%), 11 (8.59%), 8 (6.25%), 8 (6.25%), 8 (6.25%), 7 (5.47%), and 6 (4.69%) isolates respectively. Serotypes O117:H2 and O22:H8 were identified in 4 isolates each. Five serotypes contained 3 isolates each and seven serotypes contained 2 isolates each. Fifteen serotypes contained only 1 isolate each (**Table 3**).

**Table 3:** Serotypes and virulence factors of Shiga toxin-producing *Escherichia coli* isolates from yaks*.

Serotype	No. of isolates	*stx*		intimin gene	other putative adherence genes				Other virulence -associated genes				Plasmid genes	
		*stx*_1_	*stx*_2_	*eaeA*	*iha*	*efa1*	*saa*	*paa*	*cnf1*	*cnf2*	*astA*	*subA*	*ehxA*	*espP*
O2:H21	1	0	1	0	1	0	1	0	0	0	0	1	1	1
O2:H45	14	14	0	0	0	0	0	0	0	0	14	0	0	0
O6:H14	2	0	2	0	2	0	2	0	0	0	0	2	2	2
O6:H21	2	2	0	0	2	0	2	0	0	0	0	0	2	0
O8:H2	1	0	1	0	1	0	1	0	0	0	0	1	1	0
O8:H9	8	8	8	0	8	0	8	0	0	0	0	0	8	8
O8:H16	14	0	14	0	14	0	0	0	0	0	1	0	0	0
O8:H19	3	3	0	0	3	0	3	0	0	0	0	3	3	3
O8:H45	3	0	3	0	0	0	0	1	0	0	1	0	0	0
O12:H12	1	1	0	0	1	0	1	0	0	0	0	0	1	1
O12:H44	1	0	1	0	0	0	0	0	0	0	1	0	0	0
O22:H8	4	0	4	0	0	0	0	0	0	0	0	0	0	0
O52:H2	3	0	3	0	2	0	0	0	1	1	0	0	0	0
O66:H8	1	1	0	0	1	0	1	0	0	0	0	1	1	1
O66:H21	2	0	2	0	2	0	2	0	0	0	0	2	2	2
O78:H8	8	2	8	0	8	0	8	0	0	0	0	8	8	1
O78:H21	3	0	3	1	3	1	3	0	0	0	0	3	3	3
O78:H44	1	0	1	0	0	0	0	1	0	0	0	0	0	0
O78:H45	6	0	6	0	1	0	1	5	0	0	5	1	1	1
O117:H2	4	0	4	0	4	0	0	0	0	0	0	0	0	0
O117:H21	11	0	11	0	0	0	0	0	11	11	1	0	0	0
O123:H8	1	1	1	0	1	0	1	0	0	0	0	1	1	1
O127:H8	1	1	1	0	1	0	1	0	0	0	0	1	1	1
O137:H21	1	1	1	0	1	0	1	0	0	0	0	0	1	0
O149:H45	2	0	2	0	2	0	2	0	0	0	0	2	2	0
O158:H8	2	0	2	0	2	0	2	0	0	0	0	2	2	0
O158:H16	1	0	1	0	1	0	0	0	0	0	0	0	0	0
O165:H8	3	3	1	0	3	0	3	0	0	0	0	3	3	3
O165:H21	1	1	0	0	1	0	1	0	0	0	0	0	1	0
Ont/Hnt**	23	15	14	1	22	1	22	0	1	1	1	13	23	8
Total	128	53	95	2	87	2	66	7	13	13	24	44	66	36

* None of the 128 isolates were positive for *lpfA*_O157/OI-154_, *lpfA*_O157/OI-141_, *lpfA*_O113_, *katP* or *toxB* positive.

** Ont/Hnt: O or H are not typable, including Ont:H8 (8 isolates), Ont:H21 (7 isolates), Ont:H44 (2 isolates), Ont:Hnt (2 isolates), Ont:H7 (1 isolate), Ont:H40 (1 isolate), O78:Hnt (1 isolate), O6:Hnt (1 isolate).

### Presence of *stx* Genes and *stx* Subtypes and Other Virulence Factor Genes

Among the 128 STEC isolates, 33 were tested positive for *stx*_1_ only, 75 for *stx*_2_ only and 20 positive for both *stx*_1_ and *stx*_2_ (**Table 4** and **Table S1**). All of the 53 *stx*_1_-positive STEC isolates were *stx*_1a_ subtype. Of the 95 *stx*_2_-positive STEC isolates, 5 *stx*_2_ subtypes were identified with 20 isolates of *stx*_2a_, 50 of *stx*_2b_, 6 of *stx*_2c_, 21 of *stx*_2d_ and 6 of *stx*_2g_. Four isolates carried both *stx*_2a_ and *stx*_2b_, and another 4 isolates carried both *stx*_2a_ and *stx*_2c_ (**Table 4** and **Table S1**).

**Table 4:** Summary of *stx* subtyping in 128 STEC isolates.

No. of isolates	*stx* subtype					
	*stx*_1a_	*stx*_2a_	*stx*_2b_	*stx*_2c_	*stx*_2d_	*stx*_2g_
44	–	–	+	–	–	–
33	+	–	–	–	–	–
15	+	–	–	–	+	–
11	–	+	–	–	–	–
6	–	–	–	–	+	–
6	–	–	–	–	–	+
4	–	+	–	+	–	–
2	+	–	+	–	–	–
2	–	+	+	–	–	–
2	–	–	–	+	–	–
2	+	+	+	–	–	–
1	+	+	–	–	–	–
128	53	20	50	6	21	6

Only two (MN1208-22 and MN1208-34) STEC isolates were *eaeA* positive. Of the 7 putative adhesin genes (*iha*, *efa1*, *lpfA*_O157/OI-141_, *lpfA*_O157/OI-154_, *lpfA*_O113_, *saa*, *paa*) screened, *iha*, *efa1*, *saa* and *paa* were present in 87 (67.97%), 2 (1.56%), 66 (51.56%), 7 (5.47%) STEC isolates respectively. The other 3 genes were not detected in any of the isolates. Seven isolates were positive for only one gene (*paa*). Sixty six isolates were positive for both *iha* and *saa*. Two isolates were positive for 4 genes (*eaeA*, *iha*, *efa1*and *saa*). Thirty four isolates were negative for all the adherence –associated genes tested.

Four additional virulence-associated genes (*cnf1*, *cnf2*, *astA*, *subA*) were screened. Thirteen (10.16%) STEC isolates were positive for both *cnf1* and *cnf2*. Twenty four (18.75%) and 44 (34.38%) were positive for *astA* and *subA* respectively. Interestingly, the *subA* gene was present in none of STEC isolates that carried *astA* gene (**Table 3** and **Table S1**). Among the four virulence plasmid genes (*exhA*, *katP*, *espP*, *toxB*) tested, *exhA* and *espP* were present in 66 (51.56%) and 36 (28.13%) STEC isolates respectively. *espP* positive isolates also carried *exhA*. None of the 128 isolates were *katP* or *toxB* positive.

### PFGE

**Figure 1:**
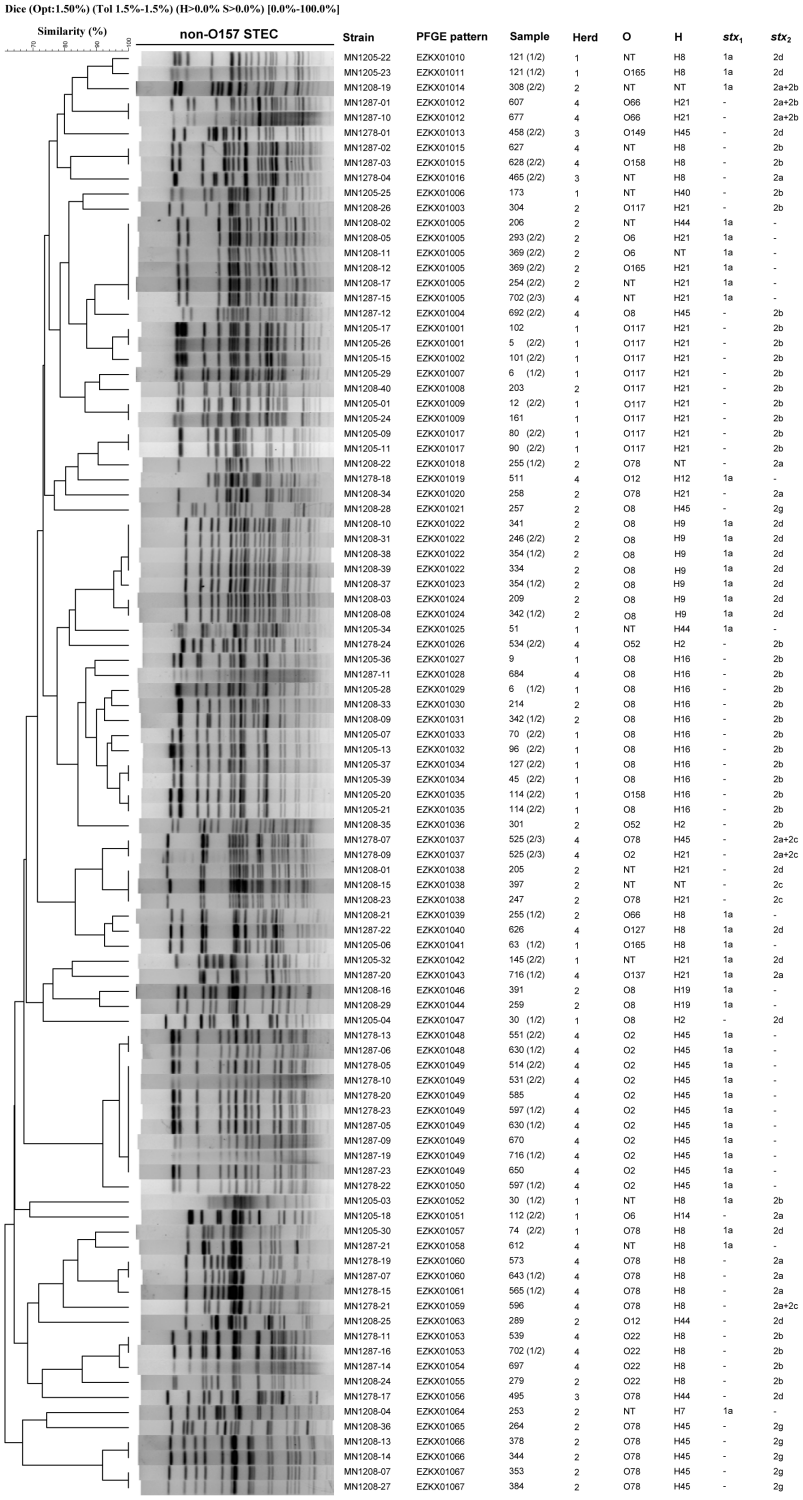
PFGE profiles of non-O157 STEC isolates from the yaks. The corresponding isolate names, PFGE patterns, no. of samples and herds, serotypes and *stx*_1_ and/or *stx*_2_ subtypes are listed on the right. For samples with more than 1 isolates, the numbers (x/y) in brackets in the sample column are number of strains (x) belonging to that PFGE pattern out of the total number of y isolates from that sample. Note that 5 isolates failed to produce a PFGE pattern and were not on the tree. For full list of isolates and their PFGE patterns and other data, see Table S1.

The 128 non-O157 STEC isolates were analyzed by PFGE to investigate their genetic relationship. Five isolates failed to produce distinctive patterns. The remaining 123 isolates were divided into 67 PFGE patterns (EZKX01001 to EZKX01067) (**Figure 1** and **Table S1**). For the 41 fecal samples with two or three isolates, the multiple isolates for 28 samples showed identical PFGE banding pattern, serotype and virulence gene profile (**Figure 1** and **Table S1**), suggesting that the multiple isolates from the same sample belong to the same STEC strain. However, 10 fecal samples contained isolates with different PFGE patterns including 3 samples (samples 354, 597, and 630) showing different PFGE patterns only (all have the same serotype), 2 samples (samples 6 and 121) showing different PFGE patterns as well as different serotypes, and 5 samples (samples 30, 255, 342, 702 and 716) showing different PFGE patterns, different serotypes and different *stx* subtypes (**Figure 1** and **Table S1**). These data suggest that some yaks were colonized by more than one STEC strain. There were also 3 samples (samples 114, 369, 525) with multiple isolates having the same PFGE type but different serotypes (**Figure 1** and **Table S1**).

The PFGE patterns were used to construct an UPGMA dendrogram (**Figure 1**) which shows that the STEC isolates were genetically diverse with nodes linking single isolates or groups of isolates at less than 80% similarity. Interestingly many isolates were grouped together with similarity at 90% or greater suggesting close genetic relationships. In particular, isolates of the same serotype had the tendency to cluster together and also carried identical *stx* or *stx* subtypes. These includes O117:H21, O8:H9, O8:H16, O2:H45, O22:H8 and O78:H45 carrying *stx*_2b_, *stx*_1a_+*stx*_2d_, *stx*_2b_, *stx*_1a_, *stx*_2b_ and *stx*_2g_ respectively. The O117:H21 isolates were in 3 related nodes while the others were in their own single node. Isolates showing identical PFGE patterns came from the same yak herd with the exception of EZKX01005 which contained 5 isolates from herd 2 and 1 isolate from herd 4. The herd 4 isolate (MN1287-15) also shared the same H antigen, *stx*_1a_ and the presence of *iha, saa* and *ehxA* as the herd 2 isolates. However this PFGE type displayed higher heterogeneity with 2 different O and 2 different H antigens and 1 isolate also carrying *astA*. The two isolates from another 2 samples (samples 114 and 525) each showed the same PFGE type but different serotypes.

The virulence gene profiles also showed a clustered distribution but less pronounced than the serotypes and *stx* subtypes. The main nodes containing the following serotypes were uniformly positive for some of the virulence factors: O117:H21 was positive for both and only *cnf1* and *cnf2*; O2:H45 for *astA* only, O8:H9 for *iha, saa,ehxA* and *espA*; O8:H16 for *iha* only; O78:H8 for *iha, saa, subA* and *ehxA*; and O78:H45 for both *paa* and *astA*. Interestingly O22:H8 carried none of the virulence factors tested (**Table S1**).

## Discussion

Ruminants, especially cattle, are the major reservoirs of STEC. The prevalence of STEC in beef cattle ranged from 0.2 to 27.8% for O157 STEC, and 2.1 to 70.1% for non-O157 STEC [38]. Bandyopadhyay *et al*. [39] recovered 42 STEC isolates from 273 rectal swab samples (15.38%) in an STEC study of the yak *Poephagus grunniens*. Our results showed a similar rate of STEC isolation. Of the 728 yak (*Bos grunniens*) fecal samples screened, 18.96% of the samples (138/728) were positive for the *stx* genes by PCR and 11.68% (85/728) by culture. Interestingly nearly 40% of the STEC positive samples by PCR were negative by culture. It seems that either the STEC cell numbers were low in the fecal sample or *stx*-positive non-*E. coli* was present in the feces. Bosilevac *et al*. [34] screened ground beef samples for STEC and only recovered STEC by culture from 300 out of the 1006 *stx*-positive samples with a success rate of just 30%.

More than 435 STEC serotypes have been recovered from cattle. Serotypes O8:H2, O8:H9, O8:H16, O8∶19, O22:H8, O117:H2, O165:H8 found in our study were also reported in cattle, beef, meat and milk product [34], [40], [41], [42]. Three serotypes, O8:H2, O8:H19 and O22:H8, have been isolated from human infections [40]. The more common HUS-causing serotypes, such as O157:H7, O26:H11, O103:H2, O111:NM, O121:H19 and O145:NM [43] were not isolated from the yaks. Neither was STEC O104:H4, the cause of the 2011 outbreaks in Germany and France. This finding is in agreement with the failure to find this isolate in cattle [44], [45] and gives an additional evidence that ruminants are not reservoirs of the outbreak isolate.

Bandyopadhyay *et al*. reported the occurrence and characteristics of STEC from feces, milk and milk products of *Poephagus grunniens*, another species of the yaks, in India [39], [46], [47]. The STEC strain isolated from the feces of *Poephagus grunniens* belonged to 22 divergent O serogroups [39]. Among these serogroups, only three (O2, O22 and O158) were also present in our study, suggesting that there are diverse STEC strains of overlapping O serogroups present in these two species of the yaks.

Since the carriage of combinations of the *stx* genes and *stx* subtypes has been associated with disease severity, the profile of the *stx* genes gives us an overview of the pathogenic potential of these STEC isolates from the yaks. In this study, 1 *stx*_1_ subtype,5 *stx*_2_ subtypes and 12 different combinations of *stx*_1_ */stx*_2_ subtypes were found in the 128 STEC isolates analyzed (**Table 4**). Manning *et al*. found that clade 8 O157:H7 strains which were significantly more likely to infect patients with HUS and more likely to have both the *stx*_2a_ and *stx*_2c_ genes, implying that the carriage of both the Stx2a and Stx2c phages contributes in part to the greater virulence of clade 8 strains [48]. Mellmann *et al*. analyzed a collection of 524 EHEC isolated from HUS patients and found that 169 (32.3%) belonged to 34 non-O157 serotypes and that profiles of *stx*_1a_ only, *stx*_1c_ only, *stx*_1a_+*stx*_2a_, *stx*_1c_+*stx*_2a_, *stx*_1c_+*stx*_2d_, *stx*_2a_ only, *stx*_2c_ only, *stx*_2d_ only and *stx*_2a_+*stx*_2c_ were present in these non-O157 STEC isolates [2]. In our STEC isolates, several of above profiles were also present including *stx*_1a_ only, *stx*_1a_+*stx*_2a_, *stx*_2a_, *stx*_2c_ only, *stx*_2d_ only and *stx*_2a_ *+stx*_2c_. These results suggest that the non-O157 STEC isolates from the yaks have the potential to cause human illness and there is a need to monitor the local human population for STEC infections.

Non–O157 STEC isolates that carry both *stx*_2_ and *eae* genes were more often associated with severe disease [49], [50]. In this study only 2 *eae* positive isolates (MN1208-22 and MN1208-34) were isolated from the yaks, both of which carried *stx*_2a,_ indicating their virulence potential. The 2 isolates were on the same PFGE node, but interspersed by other *eae* negative isolates (**Figure 1**), suggesting independent acquisition of the LEE locus by the two isolates. However, although *eae* encoded on the LEE pathogenicity island is absent from almost all of the STEC isolates, non-LEE-encoded effector proteins potentially involved in virulence have been demonstrated in some serotypes identified in this study [51].

Since the majority of the STEC isolates were *eae* negative, we investigated other factors associated with adherence including Iha and Saa, both of which have been reported to be correlated with increased adherence in the *eae* negative strains [23], [26], [52]. We found that a high proportion of the yak STEC isolates contained *iha* (67.97%) and *saa* (51.56%). This finding is similar to that reported by Bosilevac *et al*. [34] in the cattle where 88% and 73% of the STEC isolates were positive for *iha* and *saa* respectively. The study of Bosilevac *et al*. further found that the *saa* gene was always present in the absence of *eae* and was more often present in isolates that were also positive for the large virulence plasmid [34]. Our yak STEC isolates also showed a similar correlation. Interestingly our two *eae* positive isolates also harbored the *iha* and *saa* genes.

Of the two other adhesin genes, *lpfA* and *paa* screened, all 128 isolates were negative for *lpfA* while only 7 isolates were positive for *paa*, 5 of which belonged to the same serotype (O78:H45). Thirty four isolates were negative for all the adherence –associated genes tested (*eae*, *iha*, *efa1*, *lpfA*_O157/OI-154_, *lpfA*_O157/OI-141_, *lpfA*_O113_, *saa*, *paa* and *toxB*), suggesting that other novel adherence genes must exist in these STEC isolates, which warrants further investigation.

Of the additional putative virulence factors (*cnf1*, *cnf2*, *astA*, *subA*) screened, the prevalence of *cnf1* and *cnf2* was low (10.16%), with both being present in the same 13 STEC isolates. The *astA* gene was also present at a low frequency of 18.75%. However, the *subA* gene was more prevalent with 34.38% isolates positive. Interestingly, the *subA* gene was present in none of STEC isolates that harbored the *astA* gene. The significance of this mutually exclusive presence is unknown. The prevalence of *subA* in the yaks is similar to that observed in some cattle populations [53]. In contrast, a much smaller proportion of the STEC isolates isolated from human infections were positive for the *subAB* genes, which ranged from 2% in the USA to 10% in Australia [54].

Of the 4 60-MDa large plasmid encoded virulence genes [55], *ehxA*, *katP*, *espP* and *toxB* screened, none of the STEC isolates carried *katP* and *toxB*, whereas 51.56% and 28.13% STEC isolates were positive for *ehxA* and *espP* respectively. All STEC isolates that were positive for *espP* were also positive for *ehxA*. Our findings are similar to that in the cattle population based on isolation from ground beef in which *ehxA* and *espP* were more commonly present [34].

PFGE analysis showed that there is quite high genetic diversity of the STEC in the yaks and it seems that there is no separation of the STEC population between the yak herds. Eight (O8:H16, O8:H45, O22:H8, O52:H2, O78:H8, O78:H21, O78:H45 and O117:H21) of the 36 serotypes were present in 2 different herds. The serotype data also suggested that some clones are wide spread. The yaks may harbor more than one type of STEC isolates since 11.76% (10/85) of the samples contained 2 or more isolates of different PFGE patterns.

In conclusion, this study was the first report on the occurrence and characteristics of STEC isolated from yaks (*Bos grunniens*). We isolated 128 STEC isolates of different serotypes, *stx* subtypes and virulence gene profiles from the yaks (*Bos grunniens*) from the Qinghai-Tibetan plateau, revealing that *Bos grunniens* are natural reservoirs of STEC. This study further extends our knowledge of the genetic diversity and reservoir host range of STEC. The serotypes and *stx* subtypes identified were partially reported in human infections, pointing to the potential of these STEC isolates to cause disease in humans. Further investigations are needed to assess their public health significance in Tibetans and other nomadic pastoralists in this region.

## Supporting Information

Table S1 **Profiles of the 128 STEC isolates.**
(XLS)

## References

[1] Coombes BK, Wickham ME, Mascarenhas M, Gruenheid S, Finlay BB, et al (2008) Molecular analysis as an aid to assess the public health risk of non-O157 Shiga toxin-producing *Escherichia coli* strains. Appl Environ Microbiol 74: 2153–2160. 18245257 10.1128/AEM.02566-07 PMC2292595

[2] Mellmann A, Bielaszewska M, Kock R, Friedrich AW, Fruth A, et al (2008) Analysis of collection of hemolytic uremic syndrome-associated enterohemorrhagic *Escherichia coli* . Emerg Infect Dis 14: 1287–1290. 18680658 10.3201/eid1408.071082 PMC2600372

[3] Riley LW, Remis RS, Helgerson SD, McGee HB, Wells JG, et al (1983) Hemorrhagic colitis associated with a rare *Escherichia coli* serotype. N Engl J Med 308: 681–685. 6338386 10.1056/NEJM198303243081203

[4] Rangel JM, Sparling PH, Crowe C, Griffin PM, Swerdlow DL (2005) Epidemiology of *Escherichia coli* O157:H7 outbreaks, United States, 1982–2002. Emerg Infect Dis 11: 603–609. 15829201 10.3201/eid1104.040739 PMC3320345

[5] Grant J, Wendelboe AM, Wendel A, Jepson B, Torres P, et al (2008) Spinach-associated *Escherichia coli* O157:H7 outbreak, Utah and New Mexico, 2006. Emerg Infect Dis 14: 1633–1636. 18826833 10.3201/eid1410.071341 PMC2609868

[6] Michino H, Araki K, Minami S, Takaya S, Sakai N, et al (1999) Massive outbreak of *Escherichia coli* O157:H7 infection in schoolchildren in Sakai City, Japan, associated with consumption of white radish sprouts. Am J Epidemiol 150: 787–796. 10522649 10.1093/oxfordjournals.aje.a010082

[7] Xiong Y, Wang P, Lan R, Ye C, Wang H, et al (2012) A novel *Escherichia coli* O157:H7 clone causing a major hemolytic uremic syndrome outbreak in China. PLoS One 7: e36144. 22558360 10.1371/journal.pone.0036144 PMC3338595

[8] Alpers K, Werber D, Frank C, Koch J, Friedrich AW, et al (2009) Sorbitol-fermenting enterohaemorrhagic *Escherichia coli* O157:H- causes another outbreak of haemolytic uraemic syndrome in children. Epidemiol Infect 137: 389–395. 19021923 10.1017/S0950268808001465

[9] Frank C, Werber D, Cramer JP, Askar M, Faber M, et al (2011) Epidemic profile of Shiga-toxin-producing *Escherichia coli* O104:H4 outbreak in Germany. N Engl J Med 365: 1771–1780. 21696328 10.1056/NEJMoa1106483

[10] Bielaszewska M, Mellmann A, Zhang W, Kock R, Fruth A, et al (2011) Characterisation of the *Escherichia coli* strain associated with an outbreak of haemolytic uraemic syndrome in Germany, 2011: a microbiological study. Lancet Infect Dis 11: 671–676. 21703928 10.1016/S1473-3099(11)70165-7

[11] Gault G, Weill FX, Mariani-Kurkdjian P, Jourdan-da Silva N, King L, et al. (2011) Outbreak of haemolytic uraemic syndrome and bloody diarrhoea due to *Escherichia coli* O104:H4, south-west France, June 2011. Euro Surveill 16. 10.2807/ese.16.26.19905-en 21749817

[12] Jourdan-da Silva N, Watrin M, Weill FX, King LA, Gouali M, et al. (2012) Outbreak of haemolytic uraemic syndrome due to Shiga toxin-producing *Escherichia coli* O104:H4 among French tourists returning from Turkey, September 2011. Euro Surveill 17. 10.2807/ese.17.04.20065-en 22297137

[13] Ray PE, Liu XH (2001) Pathogenesis of Shiga toxin-induced hemolytic uremic syndrome. Pediatr Nephrol 16: 823–839. 11605791 10.1007/s004670100660

[14] Scheutz F, Teel LD, Beutin L, Piérard D, Buvens G, et al (2012) Multicenter evaluation of a sequence-based protocol for subtyping Shiga toxins and standardizing Stx nomenclature. J Clin Microbiol 50: 2951–2963. 22760050 10.1128/JCM.00860-12 PMC3421821

[15] Bauwens A, Bielaszewska M, Kemper B, Langehanenberg P, von Bally G, et al (2011) Differential cytotoxic actions of Shiga toxin 1 and Shiga toxin 2 on microvascular and macrovascular endothelial cells. Thromb Haemost 105: 515–528. 21136010 10.1160/TH10-02-0140

[16] Eklund M, Leino K, Siitonen A (2002) Clinical *Escherichia coli* strains carrying *stx* genes: *stx* variants and *stx*-positive virulence profiles. J Clin Microbiol 40: 4585–4593. 12454157 10.1128/JCM.40.12.4585-4593.2002 PMC154619

[17] Orth D, Grif K, Khan AB, Naim A, Dierich MP, et al (2007) The Shiga toxin genotype rather than the amount of Shiga toxin or the cytotoxicity of Shiga toxin *in vitro* correlates with the appearance of the hemolytic uremic syndrome. Diagn Microbiol Infect Dis 59: 235–242. 17931818 10.1016/j.diagmicrobio.2007.04.013

[18] Knust Z, Schmidt G (2010) Cytotoxic Necrotizing Factors (CNFs)-A growing toxin family. Toxins (Basel) 2: 116–127. 22069550 10.3390/toxins2010116 PMC3206620

[19] Savarino SJ, Fasano A, Watson J, Martin BM, Levine MM, et al (1993) Enteroaggregative *Escherichia coli* heat-stable enterotoxin 1 represents another subfamily of *E. coli* heat-stable toxin. Proc Natl Acad Sci U S A 90: 3093–3097. 8385356 10.1073/pnas.90.7.3093 PMC46243

[20] Buvens G, Lauwers S, Piérard D (2010) Prevalence of subtilase cytotoxin in verocytotoxin-producing *Escherichia coli* isolated from humans and raw meats in Belgium. Eur J Clin Microbiol Infect Dis 29: 1395–1399. 20680367 10.1007/s10096-010-1014-z

[21] Paton AW, Srimanote P, Talbot UM, Wang H, Paton JC (2004) A new family of potent AB(5) cytotoxins produced by Shiga toxigenic *Escherichia coli* . J Exp Med 200: 35–46. 15226357 10.1084/jem.20040392 PMC2213318

[22] Schmidt MA (2010) LEEways: tales of EPEC, ATEC and EHEC. Cell Microbiol 12: 1544–1552. 20716205 10.1111/j.1462-5822.2010.01518.x

[23] Tarr PI, Bilge SS, Vary JC Jr, Jelacic S, Habeeb RL, et al (2000) Iha: a novel *Escherichia coli* O157:H7 adherence-conferring molecule encoded on a recently acquired chromosomal island of conserved structure. Infect Immun 68: 1400–1407. 10678953 10.1128/iai.68.3.1400-1407.2000 PMC97294

[24] Nicholls L, Grant TH, Robins-Browne RM (2000) Identification of a novel genetic locus that is required for *in vitro* adhesion of a clinical isolate of enterohaemorrhagic *Escherichia coli* to epithelial cells. Mol Microbiol 35: 275–288. 10652089 10.1046/j.1365-2958.2000.01690.x

[25] Torres AG, Giron JA, Perna NT, Burland V, Blattner FR, et al (2002) Identification and characterization of *lpfABCC'DE*, a fimbrial operon of enterohemorrhagic *Escherichia coli* O157:H7. Infect Immun 70: 5416–5427. 12228266 10.1128/IAI.70.10.5416-5427.2002 PMC128367

[26] Paton AW, Srimanote P, Woodrow MC, Paton JC (2001) Characterization of Saa, a novel autoagglutinating adhesin produced by locus of enterocyte effacement-negative Shiga-toxigenic *Escherichia coli* strains that are virulent for humans. Infect Immun 69: 6999–7009. 11598075 10.1128/IAI.69.11.6999-7009.2001 PMC100080

[27] Batisson I, Guimond MP, Girard F, An H, Zhu C, et al (2003) Characterization of the novel factor Paa involved in the early steps of the adhesion mechanism of attaching and effacing *Escherichia coli* . Infect Immun 71: 4516–4525. 12874331 10.1128/IAI.71.8.4516-4525.2003 PMC166039

[28] Brunder W, Schmidt H, Frosch M, Karch H (1999) The large plasmids of Shiga-toxin-producing *Escherichia coli* (STEC) are highly variable genetic elements. Microbiology 145 (Pt 5): 1005–1014. 10.1099/13500872-145-5-1005 10376815

[29] Oporto B, Esteban JI, Aduriz G, Juste RA, Hurtado A (2008) *Escherichia coli* O157:H7 and non-O157 Shiga toxin-producing *E. coli* in healthy cattle, sheep and swine herds in Northern Spain. Zoonoses Public Health 55: 73–81. 18234025 10.1111/j.1863-2378.2007.01080.x

[30] Bentancor A, Rumi MV, Carbonari C, Gerhardt E, Larzabal M, et al (2012) Profile of Shiga toxin-producing *Escherichia coli* strains isolated from dogs and cats and genetic relationships with isolates from cattle, meat and humans. Vet Microbiol 156: 336–342. 22119188 10.1016/j.vetmic.2011.10.030

[31] Sanchez S, Martinez R, Rey J, Garcia A, Blanco J, et al (2010) Pheno-genotypic characterisation of *Escherichia coli* O157:H7 isolates from domestic and wild ruminants. Vet Microbiol 142: 445–449. 19914011 10.1016/j.vetmic.2009.10.009

[32] Eggert M, Stuber E, Heurich M, Fredriksson-Ahomaa M, Burgos Y, et al. (2012) Detection and characterization of Shiga toxin-producing *Escherichia coli* in faeces and lymphatic tissue of free-ranging deer. Epidemiol Infect: 1–9. 10.1017/S0950268812000246 PMC9152051 22370185

[33] Sanchez S, Martinez R, Garcia A, Vidal D, Blanco J, et al (2010) Detection and characterisation of O157:H7 and non-O157 Shiga toxin-producing *Escherichia coli* in wild boars. Vet Microbiol 143: 420–423. 20005055 10.1016/j.vetmic.2009.11.016

[34] Bosilevac JM, Koohmaraie M (2011) Prevalence and characterization of non-O157 shiga toxin-producing *Escherichia coli* isolates from commercial ground beef in the United States. Appl Environ Microbiol 77: 2103–2112. 21257806 10.1128/AEM.02833-10 PMC3067332

[35] Qiu Q, Zhang G, Ma T, Qian W, Wang J, et al (2012) The yak genome and adaptation to life at high altitude. Nat Genet 44: 946–949. 22751099 10.1038/ng.2343

[36] Huang XD, Tan HY, Long R, Liang JB, Wright AD (2012) Comparison of methanogen diversity of yak (*Bos grunniens*) and cattle (*Bos taurus*) from the Qinghai-Tibetan plateau, China. BMC Microbiol 12: 237. 23078429 10.1186/1471-2180-12-237 PMC3502369

[37] DebRoy C, Roberts E, Fratamico PM (2011) Detection of O antigens in *Escherichia coli* . Anim Health Res Rev 12: 169–185. 22152292 10.1017/S1466252311000193

[38] Hussein HS, Bollinger LM (2005) Prevalence of Shiga toxin-producing *Escherichia coli* in beef cattle. J Food Prot 68: 2224–2241. 16245735 10.4315/0362-028x-68.10.2224

[39] Bandyopadhyay S, Lodh C, Sarkar M, Ghosh MK, Bera AK, et al (2012) Prevalence, molecular fingerprinting and drug resistance profile of enterovirulent *Escherichia coli* isolates from free-ranging yaks of Tawang district, Arunachal Pradesh, India. Trop Anim Health Prod 44: 1063–1072. 22228494 10.1007/s11250-011-0041-9

[40] Hussein HS (2007) Prevalence and pathogenicity of Shiga toxin-producing *Escherichia coli* in beef cattle and their products. J Anim Sci 85: E63–72. 17060419 10.2527/jas.2006-421

[41] Martin A, Beutin L (2011) Characteristics of Shiga toxin-producing *Escherichia coli* from meat and milk products of different origins and association with food producing animals as main contamination sources. Int J Food Microbiol 146: 99–104. 21371769 10.1016/j.ijfoodmicro.2011.01.041

[42] Hauser E, Mellmann A, Semmler T, Stoeber H, Wieler LH, et al (2013) Phylogenetic and Molecular Analysis of Food-Borne Shiga Toxin-Producing *Escherichia coli* . Appl Environ Microbiol 79: 2731–2740. 23417002 10.1128/AEM.03552-12 PMC3623172

[43] Karmali MA, Mascarenhas M, Shen S, Ziebell K, Johnson S, et al (2003) Association of genomic O island 122 of *Escherichia coli* EDL 933 with verocytotoxin-producing *Escherichia coli* seropathotypes that are linked to epidemic and/or serious disease. J Clin Microbiol 41: 4930–4940. 14605120 10.1128/JCM.41.11.4930-4940.2003 PMC262514

[44] Wieler LH, Semmler T, Eichhorn I, Antao EM, Kinnemann B, et al (2011) No evidence of the Shiga toxin-producing *E. coli* O104:H4 outbreak strain or enteroaggregative *E. coli* (EAEC) found in cattle faeces in northern Germany, the hotspot of the 2011 HUS outbreak area. Gut Pathog 3: 17. 22051440 10.1186/1757-4749-3-17 PMC3227623

[45] Auvray F, Dilasser F, Bibbal D, Kerouredan M, Oswald E, et al (2012) French cattle is not a reservoir of the highly virulent enteroaggregative Shiga toxin-producing *Escherichia coli* of serotype O104:H4. Vet Microbiol 158: 443–445. 22424867 10.1016/j.vetmic.2012.02.029

[46] Bandyopadhyay S, Biswas TK, Sasmal D, Ghosh MK, Dutta TK, et al (2009) Virulence gene and antibiotic resistance profile of Shiga-toxin-producing *Escherichia coli* prevalent in captive yaks (*Poephagus grunniens*). Vet Microbiol 138: 403–404. 19477084 10.1016/j.vetmic.2009.04.016

[47] Bandyopadhyay S, Lodh C, Rahaman H, Bhattacharya D, Bera AK, et al (2012) Characterization of shiga toxin producing (STEC) and enteropathogenic *Escherichia coli* (EPEC) in raw yak (*Poephagus grunniens*) milk and milk products. Res Vet Sci 93: 604–610. 22226073 10.1016/j.rvsc.2011.12.011

[48] Manning SD, Motiwala AS, Springman AC, Qi W, Lacher DW, et al (2008) Variation in virulence among clades of *Escherichia coli* O157:H7 associated with disease outbreaks. Proc Natl Acad Sci U S A 105: 4868–4873. 18332430 10.1073/pnas.0710834105 PMC2290780

[49] Boerlin P, McEwen SA, Boerlin-Petzold F, Wilson JB, Johnson RP, et al (1999) Associations between virulence factors of Shiga toxin-producing *Escherichia coli* and disease in humans. J Clin Microbiol 37: 497–503. 9986802 10.1128/jcm.37.3.497-503.1999 PMC84443

[50] Werber D, Fruth A, Buchholz U, Prager R, Kramer MH, et al (2003) Strong association between shiga toxin-producing *Escherichia coli* O157 and virulence genes *stx* _2_ and *eae* as possible explanation for predominance of serogroup O157 in patients with haemolytic uraemic syndrome. Eur J Clin Microbiol Infect Dis 22: 726–730. 14614596 10.1007/s10096-003-1025-0

[51] Creuzburg K, Middendorf B, Mellmann A, Martaler T, Holz C, et al (2011) Evolutionary analysis and distribution of type III effector genes in pathogenic *Escherichia coli* from human, animal and food sources. Environ Microbiol 13: 439–452. 20880329 10.1111/j.1462-2920.2010.02349.x

[52] Jenkins C, Perry NT, Cheasty T, Shaw DJ, Frankel G, et al (2003) Distribution of the *saa* gene in strains of Shiga toxin-producing *Escherichia coli* of human and bovine origins. J Clin Microbiol 41: 1775–1778. 12682185 10.1128/JCM.41.4.1775-1778.2003 PMC153935

[53] Irino K, Vieira MA, Gomes TA, Guth BE, Naves ZV, et al (2010) Subtilase cytotoxin-encoding *subAB* operon found exclusively among Shiga toxin-producing *Escherichia coli* strains. J Clin Microbiol 48: 988–990. 20089761 10.1128/JCM.00010-10 PMC2832406

[54] Wolfson JJ, Jandhyala DM, Gorczyca LA, Qadeer Z, Manning SD, et al (2009) Prevalence of the operon encoding subtilase cytotoxin in non-O157 Shiga toxin-producing *Escherichia coli* isolated from humans in the United States. J Clin Microbiol 47: 3058–3059. 19571032 10.1128/JCM.00706-09 PMC2738072

[55] Newton HJ, Sloan J, Bulach DM, Seemann T, Allison CC, et al (2009) Shiga toxin-producing *Escherichia coli* strains negative for locus of enterocyte effacement. Emerg Infect Dis 15: 372–380. 19239748 10.3201/eid1502.080631 PMC2681110

[56] Paton AW, Beutin L, Paton JC (1995) Heterogeneity of the amino-acid sequences of *Escherichia coli* Shiga-like toxin type-I operons. Gene 153: 71–74. 7883188 10.1016/0378-1119(94)00777-p

[57] Brandal LT, Lindstedt BA, Aas L, Stavnes TL, Lassen J, et al (2007) Octaplex PCR and fluorescence-based capillary electrophoresis for identification of human diarrheagenic *Escherichia coli* and *Shigella* spp. J Microbiol Methods 68: 331–341. 17079041 10.1016/j.mimet.2006.09.013

[58] Schmidt H, Zhang WL, Hemmrich U, Jelacic S, Brunder W, et al (2001) Identification and characterization of a novel genomic island integrated at *selC* in locus of enterocyte effacement-negative, Shiga toxin-producing *Escherichia coli* . Infect Immun 69: 6863–6873. 11598060 10.1128/IAI.69.11.6863-6873.2001 PMC100065

[59] Toma C, Martinez Espinosa E, Song T, Miliwebsky E, Chinen I, et al (2004) Distribution of putative adhesins in different seropathotypes of Shiga toxin-producing *Escherichia coli* . J Clin Microbiol 42: 4937–4946. 15528677 10.1128/JCM.42.11.4937-4946.2004 PMC525252

[60] Szalo IM, Goffaux F, Pirson V, Pierard D, Ball H, et al (2002) Presence in bovine enteropathogenic (EPEC) and enterohaemorrhagic (EHEC) *Escherichia coli* of genes encoding for putative adhesins of human EHEC strains. Res Microbiol 153: 653–658. 12558184 10.1016/s0923-2508(02)01379-7

[61] Doughty S, Sloan J, Bennett-Wood V, Robertson M, Robins-Browne RM, et al (2002) Identification of a novel fimbrial gene cluster related to long polar fimbriae in locus of enterocyte effacement-negative strains of enterohemorrhagic *Escherichia coli* . Infect Immun 70: 6761–6769. 12438351 10.1128/IAI.70.12.6761-6769.2002 PMC133005

[62] Paton AW, Paton JC (2002) Direct detection and characterization of Shiga toxigenic *Escherichia coli* by multiplex PCR for *stx* _1_, *stx* _2_, *eae*, *ehxA*, and *saa* . J Clin Microbiol 40: 271–274. 11773130 10.1128/JCM.40.1.271-274.2002 PMC120136

[63] Zweifel C, Schumacher S, Beutin L, Blanco J, Stephan R (2006) Virulence profiles of Shiga toxin 2e-producing *Escherichia coli* isolated from healthy pig at slaughter. Vet Microbiol 117: 328–332. 16872761 10.1016/j.vetmic.2006.06.017

[64] Pass MA, Odedra R, Batt RM (2000) Multiplex PCRs for identification of *Escherichia coli* virulence genes. J Clin Microbiol 38: 2001–2004. 10790141 10.1128/jcm.38.5.2001-2004.2000 PMC86652

[65] Yamamoto T, Echeverria P (1996) Detection of the enteroaggregative *Escherichia coli* heat-stable enterotoxin 1 gene sequences in enterotoxigenic *E. coli* strains pathogenic for humans. Infect Immun 64: 1441–1445. 8606115 10.1128/iai.64.4.1441-1445.1996 PMC173940

[66] Paton AW, Paton JC (2005) Multiplex PCR for direct detection of Shiga toxigenic *Escherichia coli* strains producing the novel subtilase cytotoxin. J Clin Microbiol 43: 2944–2947. 15956427 10.1128/JCM.43.6.2944-2947.2005 PMC1151958

[67] Schmidt H, Beutin L, Karch H (1995) Molecular analysis of the plasmid-encoded hemolysin of *Escherichia coli* O157:H7 strain EDL 933. Infect Immun 63: 1055–1061. 7868227 10.1128/iai.63.3.1055-1061.1995 PMC173109

[68] Brunder W, Schmidt H, Karch H (1996) KatP, a novel catalase-peroxidase encoded by the large plasmid of enterohaemorrhagic *Escherichia coli* O157:H7. Microbiology 142 (Pt 11): 3305–3315. 10.1099/13500872-142-11-3305 8969527

[69] Tarr CL, Large TM, Moeller CL, Lacher DW, Tarr PI, et al (2002) Molecular characterization of a serotype O121:H19 clone, a distinct Shiga toxin-producing clone of pathogenic *Escherichia coli* . Infect Immun 70: 6853–6859. 12438362 10.1128/IAI.70.12.6853-6859.2002 PMC133070

